# Web-Based Technology for Remote Viewing of Radiological Images: App Validation

**DOI:** 10.2196/16224

**Published:** 2020-09-25

**Authors:** Qiusha Min, Xin Wang, Bo Huang, Liangzhou Xu

**Affiliations:** 1 School of Educational Information Technology Central China Normal University Wuhan, Hubei China; 2 Department of Radiology Wuhan Hospital of Traditional Chinese Medicine Wuhan China

**Keywords:** internet access, medical informatics applications, computer-assisted image analyses, computer-assisted three-dimensional imaging, medical imaging, radiology, application

## Abstract

**Background:**

Internet technologies can create advanced and rich web-based apps that allow radiologists to easily access teleradiology systems and remotely view medical images. However, each technology has its own drawbacks. It is difficult to balance the advantages and disadvantages of these internet technologies and identify an optimal solution for the development of medical imaging apps.

**Objective:**

This study aimed to compare different internet platform technologies for remotely viewing radiological images and analyze their advantages and disadvantages.

**Methods:**

Oracle Java, Adobe Flash, and HTML5 were each used to develop a comprehensive web-based medical imaging app that connected to a medical image server and provided several required functions for radiological interpretation (eg, navigation, magnification, windowing, and fly-through). Java-, Flash-, and HTML5-based medical imaging apps were tested on different operating systems over a local area network and a wide area network. Three computed tomography colonography data sets and 2 ordinary personal computers were used in the experiment.

**Results:**

The experimental results demonstrated that Java-, Flash-, and HTML5-based apps had the ability to provide real-time 2D functions. However, for 3D, performances differed between the 3 apps. The Java-based app had the highest frame rate of volume rendering. However, it required the longest time for surface rendering and failed to run surface rendering in macOS. The HTML5-based app had the fastest surface rendering and the highest speed for fly-through without platform dependence. Volume rendering, surface rendering, and fly-through performances of the Flash-based app were significantly worse than those of the other 2 apps.

**Conclusions:**

Oracle Java, Adobe Flash, and HTML5 have individual strengths in the development of remote access medical imaging apps. However, HTML5 is a promising technology for remote viewing of radiological images and can provide excellent performance without requiring any plug-ins.

## Introduction

Recently, modern technology has made it possible to generate digital images using medical equipment. Compared with traditional film-based images, these types of images have several advantages (eg, they are easy to share, transmit, and process) [1]. These advantages promote the popularity of the digital imaging systems in hospitals all over the world, and offer the possibility for remote viewing and processing. However, the successful implementation of a teleradiology system requires a fast network and easy access [2]. If the system does not meet these requirements, radiologists may be reluctant to use the teleradiology system.

Internet technologies can create advanced and rich web-based apps that allow radiologists to easily access teleradiology systems and remotely view medical images. Compared with picture archiving and communication systems or other imaging workstations which require dedicated hardware and software, a web-based app is easy to set up and has a low cost [3]. These apps can be run on almost all personal computers without the need for powerful equipment on the client side. There are 3 major internet technologies, Oracle Java [4], Adobe Flash [5], and HTML5 [6], to create these apps. In the past few decades, these 3 technologies have been used in the field of medical imaging [7-16]; however, each technology has its own drawbacks. For example, plug-ins are required by Java and Flash. Regarding HTML5, the level of support and expected performance vary depending on the browser. Thus, it is difficult to balance the advantages and disadvantages of these internet technologies and identify an optimal solution for the development of medical imaging apps. Owing to the significant growth of teleradiology and web-based radiology subspecialty training, there is a need for quantitative and qualitative evaluations of different internet technologies in the field of medical imaging [17,18].

In this study, we used different technologies—Java (version 8; Oracle Corporation), Adobe Flash (version 32; Adobe Inc), and HTML5 (version 5.3; World Wide Web Consortium)—to develop web-based medical imaging apps. Subsequently, experiments were conducted to demonstrate the performance of these apps. Accordingly, the primary aim of this study was the evaluation of the performance of medical imaging apps developed with Oracle Java, Adobe Flash, and HTML5 in various scenarios. We also aimed to analyze the advantages and disadvantages of these technologies in the field of medical imaging. We believe these performance comparisons can guide developers in their efforts to identify suitable technologies to create web-based medical imaging apps, thus allowing radiologists to visualize and interpret images remotely, quickly, and effortlessly.

## Methods

### App Design

Medical imaging apps have several basic functions. First, the app needs to interact directly with the local file system to avoid network latency. The user can then use various 2D image processing tools, such as zooming and windowing, to identify useful information contained in the 2D image. In addition, the interpretation may be supported by 3D functions so that the volumetric data set can provide additional information on the anatomy or pathology of the patient [19]. Consequently, comprehensive medical imaging apps should meet the following requirements: (1) interact with local file systems, (2) have basic 2D image processing functions, and (3) allow 3D visualization of selected regions of interest in the data sets.

In this study, 3 demo apps for computed tomography (CT) colonography (also known as virtual colonoscopy) were developed using Oracle Java, Adobe Flash, and HTML5. These apps were designed to satisfy the aforementioned criteria, and they were used to evaluate Oracle Java, Adobe Flash, and HTML5 as tools to determine the best architecture for the development of a medical imaging app.

These apps provide remote access in such a way that radiologists can view images from a downloaded data set and manipulate them using 2D or 3D functions. They can be placed as a client component in a large teleradiology system. This study focuses solely on the client app and presents a comparison of 2D and 3D performance of the Oracle Java, Adobe Flash, and HTML5 technologies for the development of such medical imaging apps.

### Operational Flow of Radiological Interpretation Using Demo Apps

#### Step 1

Select a data set to be interpreted and click the *Download* button to activate the download process. The selected data set is now stored on the local computer.

#### Step 2

The first slice of the data set is automatically displayed on the screen in the Java-based app. In the cases of the Flash and HTML5 apps, users need to click the *Choose file* button and select in the dialog box the downloaded file in either of the 2 apps. Thereafter, the first slice of the data set is displayed.

#### Step 3

Navigate through the image data set using the *Previous* and *Next* buttons or mouse scrolling.

#### Step 4

Interpret the data set using 2D image processing tools, such as zoom in, zoom out, and windowing.

#### Step 5

Interpret the data set using 3D visualization tools, such as 3D rendering and fly-through.

### App Implementation

#### Access to the Local File System

**Figure 1:**
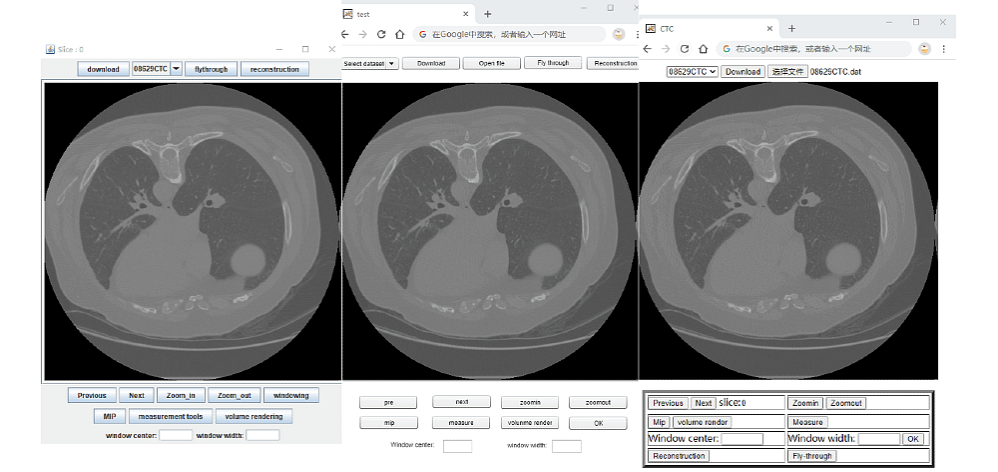
Graphical user interface used to display the first slice in the data set: (left) Java, (center) Flash, and (right) HTML5.

All 3 apps enabled the user to choose a CT colonography data set for study (Figure 1). The selected data set was then transmitted to the client and stored on the local computer using a custom data format. Currently, Oracle Java, Adobe Flash, and HTML5 use different local file reading and writing technologies. Oracle Java downloaded the file via HTTP using the Java HttpURLConnection class. This class was used to read and write the resources referenced by a URL (uniform resource locator). Once the download was completed, the RandomAccessFile class was used to read local files in the Java-based app. Adobe Flash used the FileReference class to provide a safe way to directly read and write data to the local system (provided that the action was sanctioned by the user). Using this class in the app, the study data set was stored on the local computer disk and could then be navigated easily and efficiently. HTML5 had a new input type <input type = “file”> which provided a standard way to interact with local files. After reading the downloaded file, the first slice in the data set was automatically displayed on the screen.

#### Image Processing

Another crucial requirement for these apps was pixel-level operation. For medical imaging apps, basic 2D image processing functions include magnification and windowing. For the magnification function, Java used *scaleX*() and *scaleY*() in the ScalePane class to zoom in and zoom out. Flash used the Zoom class to zoom in or out of the object. HTML5 used the canvas *drawImage*() method to zoom in and out. In terms of windowing, Java used *setRGB*() to set the pixels in BufferedImage to the specified RGB value. Flash was implemented using the BitmapData class. The *setPixel*() and *getPixel*() methods in the BitmapData class were used to change the value of each pixel in the image. For HTML5, a <canvas> element that has the ability to define the color of the pixels was used.

**Figure 2:**
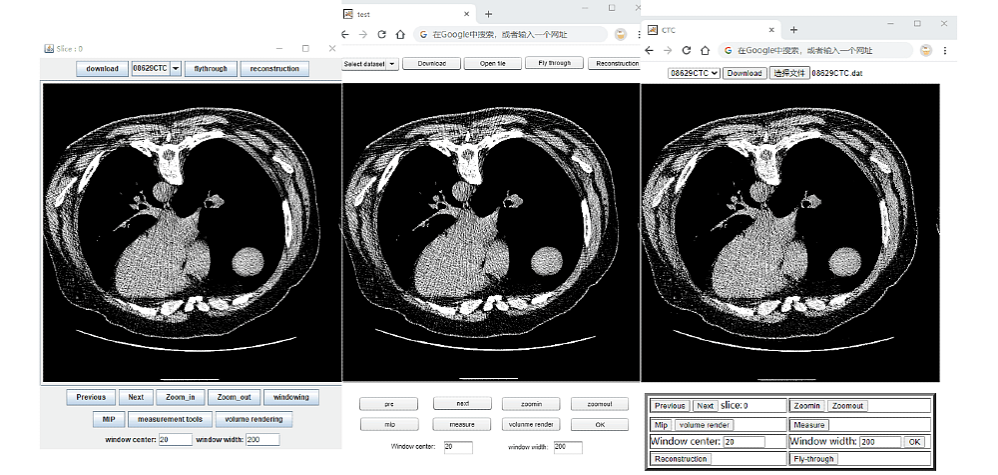
An anatomical axial slice at the thoracic level, wherein the center parameter is 20 HU, and the width is 200 HU. (left) Java, (center) Flash, and (right) HTML5. Screen resolution is 1920×1080.

**Figure 3:**
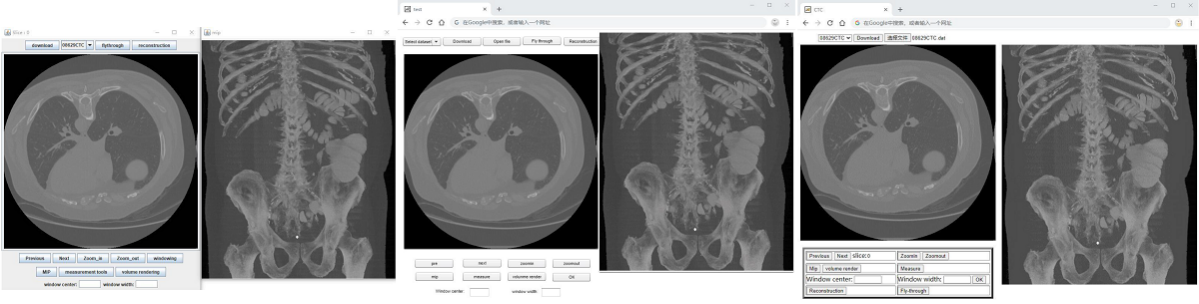
Graphical user interface and demonstration of maximum intensity projection. (left) Java, (center) Flash, and (right) HTML5.

Figure 2 depicts a series of screenshots of the user interfaces of the windowing function in the 3 tested apps. The image is a windowed slice with the following parameters: the center of the window is 20 HU (where HU is Hounsfield units) and the width of the window is 200 HU. Figure 3 is a screenshot of the user interface of maximum intensity projection in Java-, Flash- and HTML5-based apps. Measurement and magnification functions can also be implemented by these 3 apps.

#### 3D Visualization

It is well known that 3D visualization is a computationally intensive task. Hence, this work is typically implemented at a workstation equipped with a high-performance graphics processing unit (GPU). However, based on our previous research [11,13,15], it is feasible to implement 3D visualization on a personal computer using Oracle Java, Adobe Flash, or HTML5. In this implementation, 3D visualization was based on both surface rendering and volume rendering.

**Figure 4:**
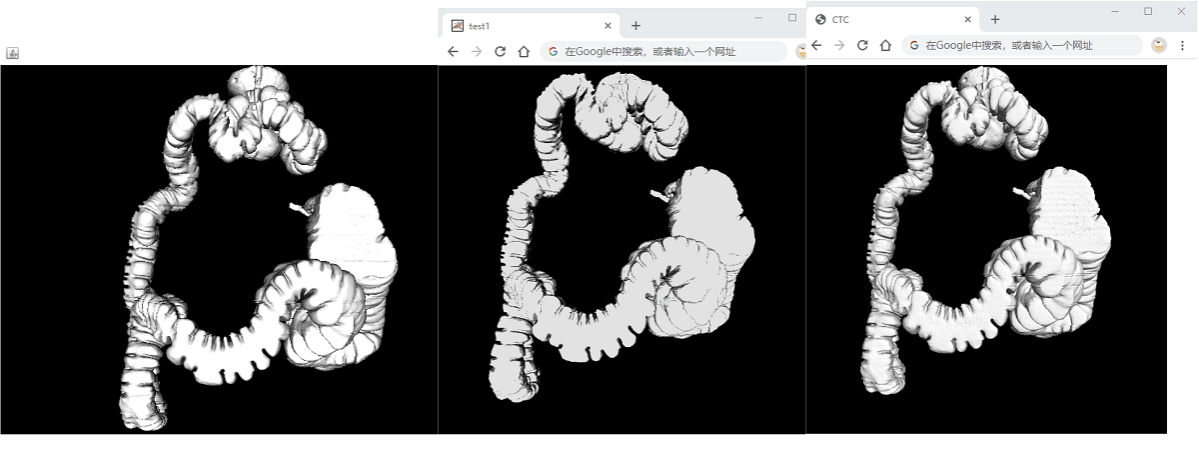
Screenshot of a 3D model of the entire colon in the browser. (left) Java, (center) Flash, and (right) HTML5.

Surface rendering generally involved 2 stages: surface extraction and 3D rendering. The marching cubes algorithm was used to extract the isosurface from a volumetric data set [20]. The information of the extracted surface (ie, the vertex and the normal) were stored on a server. Once the user sent the request to view the 3D data, the corresponding vertex and normal files were sent to the client. Subsequently, the client side was responsible for rendering the 3D model surface. Currently, Oracle Java, Adobe Flash, and HTML5 enable the provision of hardware 3D rendering, which is a fast rendering mode when compared to that of software rendering. Java used Canvas3D to implement 3D rendering. For Adobe Flash, a Context3D object was used in its 3D app programming interface (API). Using the *createVertexBuffer*() method, Flash could send the vertices and normals to the GPU directly and perform a fast reconstruction. The combination of HTML5 with WebGL could also realize fast 3D rendering. Figure 4 presents 3D colon models rendered by Java-, Flash-, and HTML5-based apps. The user could also interact with this model and perform various operations, such as rotation and translation, using the mouse.

**Figure 5:**
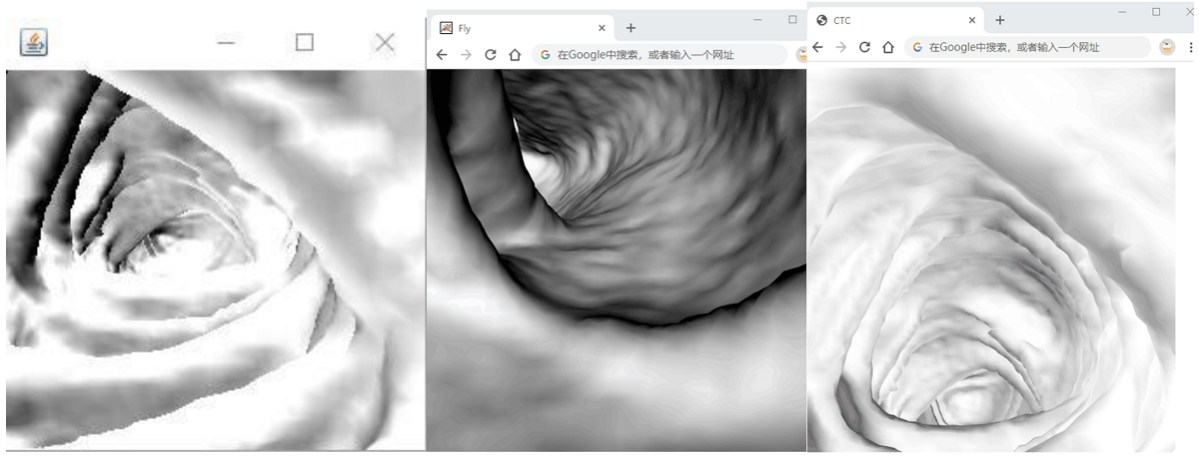
Screenshot of the implementation of the fly-through in the browser. (left) Java, (center) Flash, and (right) HTML5.

Virtual fly-through navigation is a function used to manipulate the results of 3D reconstruction. It creates a virtual camera within the colon that moves along a planned path (commonly referred to as the colon centerline [21]); the radiologist can observe the interior of the colon using the continuous movement of the camera. This advanced imaging technique can help the radiologist make more accurate judgments about the lesion. Figure 5 presents the results of different techniques for implementing 3D fly-through within the colon, running in a browser.

**Figure 6:**
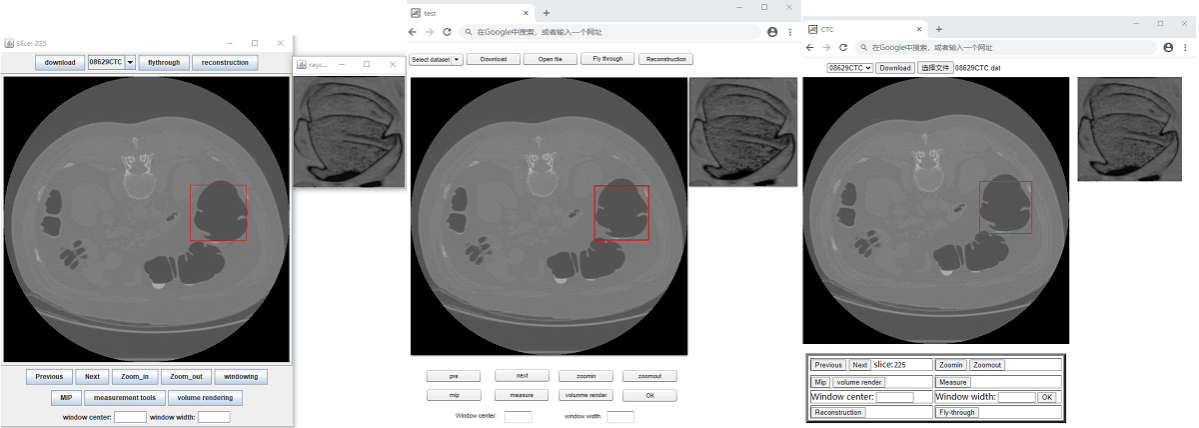
Screenshot of volume rendering in the browser. (left) Java, (center) Flash, and (right) HTML5.

Volume rendering is another type of 3D visualization that can represent the interior information of the 3D data set. Our implementation of volume rendering was based on a ray casting algorithm due to its ability to render high quality images [22]. This technique involved intensive computations resulting in low rendering speeds; however, it was feasible to define a subvolume to represent a region of interest. Volume rendering was then applied in this subvolume. In our apps, the size of the subvolume was 100×100×100 pixels and could be selected by the user. Figure 6 presents a region of interest rendered by the ray casting algorithm in Java-, Flash-, and HTML5-based apps.

### Experiment Design

#### Overview

To compare the performances of the Java-, Flash-, and HTML5-based apps, 3 types of experiments were conducted: determining the performance of the apps running on the same platform (in Windows; Experiment 1); evaluating the performance of the apps on multiple platforms (Experiment 2); comparing the performances of the apps using a local area network (LAN) or a wide area network (WAN) (Experiment 3). CT colonography data sets (n=3), which were downloaded from The Cancer Imaging Archive [23], were used. Descriptions of the data sets are presented in Table 1, and the information about the computers used in the experiments is provided in Table 2. It is evident that the computers were ordinary personal computers for regular users.

**Table 1:** CT data sets used in the experiments.

Data set	Data set size Size		3D visualization		
	kB	pixel	Vertex file (kB)	Normal file (kB)	Number of faces
1	321,536	512×512×628	57,747	57,747	1,642,580
2	312,320	512×512×610	59,545	59,545	1,693,700
3	256,000	512×512×500	60,236	60,236	1,713,372

It should be noted that while the performances of Java- and Flash-based apps are browser independent, they are dependent on Java Virtual Machine and Flash Player, respectively. However, HTML5 is solely dependent on the browser, and our previous research [15] has demonstrated that Google Chrome can provide stable and excellent HTML5 performance. Thus, Google Chrome was used to run the HTML5-based app as well as to launch the Flash- and Java-based apps in this experiment. The details of the testing metrics in this study are presented in Table 3.

**Table 2:** Computers used in the experiments.

Computer	Type	Operating system	CPU^a^	Memory	GPU^b^
1	Desktop	Windows 10, 64 bits	Intel Core i5-8400 @2.80 GHz	16.00 GB	NVIDIA GeForce GTX 1050Ti
2	Desktop	Ubuntu 20.04	Intel Core i5-8400 @2.80 GHz	16.00 GB	NVIDIA GeForce GTX 1050Ti
3	Laptop (MacBook Pro)	MacOS Sierra 10.12.5	Intel Core Intel i5 @ 2.30 GHz	8.00 GB	Intel Iris Plus Graphics 640

^a^CPU: central processing unit.

^b^GPU: graphics processing unit.

**Table 3:** Details of testing metrics of this study.

Function	Label	Description	Measurement		
			Java	Adobe Flash	HTML5
**Data access**					
	M1	Execution time for downloading a medical image data set	Java	ActionScript 3.0	Manual
**2D image processing**					
	M2	Execution time for viewing a slice in a medical image data set	Java	ActionScript 3.0	JavaScript
	M3	Execution time for implementing windowing per slice	Java	ActionScript 3.0	JavaScript
	M4	Execution time for implementing magnification per slice	Java	ActionScript 3.0	JavaScript
	M5	Execution time for implementing mouse wheel per slice	Java	ActionScript 3.0	JavaScript
	M6	Execution time for implementing measure tool per slice	Java	ActionScript 3.0	JavaScript
	M7	Execution time for implementing maximum intensity projection	Java	ActionScript 3.0	JavaScript
**3D visualization**					
	M8	Execution time for downloading the vertex and normal files of a medical image data set	Java	ActionScript 3.0	Manual
	M9	Execution time for rendering a 3D model based on the downloaded vertex and normal files	Java	ActionScript 3.0	JavaScript
	M10	Frame rate of fly-through	Java	ActionScript 3.0	JavaScript
	M11	Frame rate of software-based volume rendering	Java	ActionScript 3.0	JavaScript

#### Experiment 1

The first experiment was carried out with 3 data sets using LAN to compare the performances of different apps running on the same platform. Computer 1 was chosen to test the Java-, Flash-, and HTML5-based apps on a Windows operating system. Each function was implemented 20 times in each app.

#### Experiment 2

The second experiment was used to determine the performance consistency of different internet technologies among multiple platforms. Computers 1, 2, and 3 were used in this experiment. Therefore, Java-, Flash-, and HTML5-based apps were run on Windows, macOS, and Linux platforms, respectively. Each function in the 3 apps was implemented 20 times on these platforms using data set 1 over a LAN.

#### Experiment 3

In the third experiment, computer 1 was used to evaluate the performances of Java-, Flash-, and HTML5-based apps over the LAN and WAN. This computer was equipped with Windows, and hence all the 3 apps were tested on the same platform. All 3 data sets were used in the experiment. Java-, Flash-, and HTML5-based apps were tested based on data sets 1, 2, and 3, to determine the performance differences when they ran over a LAN and WAN. In WAN, the 3 apps accessed the medical data set and vertex and normal files on the remote server. The bandwidth of the connecting network was 50 Mbps, and it had a download speed of approximately 5.1 MB/s. The download sizes for the medical image data set, vertex file, and normal file are listed in Table 2.

Each function in the app was implemented 20 times by Java Virtual Machine (version 1.8.0), Flash Player (version 32.0.0.433), and Chrome (version 83.0.4103.97), either over the LAN or WAN.

### Radiologist Feedback

In order to collect feedback on the apps, we conducted a pilot trial. Radiologists (n=5) at Wuhan Hospital of Traditional Chinese Medicine participated in this trial. They first received a brief introduction of the project and our medical imaging apps. After that, each of them was required to interpret 10 CT examinations using the Java-based app, 10 CT examinations using the Flash-based app, and 10 CT examinations using the HTML5-based apps on a Windows personal computer on the LAN.

After the trial, they filled out questionnaires (Multimedia Appendix 1). A 5-point Likert scale was used to represent the radiologists’ opinions on a particular question or statement: strongly disagree, disagree, unsure, agree, and strongly agree. Radiologists’ responses were recorded on a 1-5 scale, with higher numbers representing stronger agreement.

## Results

### Experiment 1: General Performance

The average performances for each function are presented in Table 4 (Experiment 1). The comparison revealed that each technology had its own advantages. Java was associated with the shortest downloading time and highest frame rate for software-based volume rendering. However, it performed poorly at surface rendering. HTML5 surface rendering performed best. In terms of 2D functions, such as zooming and windowing, all 3 apps performed similarly. Overall, HTML5 outperformed the other 2 technologies, with the exception of downloading and software-based volume rendering.

**Table 4:** Comparison of the performances of the 3 apps in various scenarios.

Technology			Data access	2D image processing						3D visualization			
			M1 (s^a^)	M2 (s)	M3 (s)	M4 (s)	M5 (s)	M6 (s)	M7 (s)	M8 (s)	M9 (s)	M10 (fps^b^)	M11 (fps)
**Experiment 1**													
	**Data set 1**												
		Java (Windows)	29.87	0.0073	0.0142	0.0015	0.0077	0.0010	0.3446	10.76	271.21	32.25	1.92
		Flash (Windows)	27.76	0.0144	0.0193	0.0004	0.0146	0.0001	26.7071	10.24	16.20	10.21	0.05
		HTML5 (Windows)	28.58	0.0009	0.0133	0.0006	0.0008	0.0002	2.4036	13.46	1.09	59.30	0.56
	**Data set 2**												
		Java (Windows)	29.16	0.0066	0.0094	0.0016	0.0058	0.0010	0.3701	10.29	280.54	34.23	1.97
		Flash (Windows)	26.97	0.0148	0.0189	0.0007	0.0149	0.0002	27.5514	10.35	17.59	9.91	0.05
		HTML5 (Windows)	27.98	0.0008	0.0154	0.0004	0.0009	0.0003	2.4560	12.38	1.25	60.05	0.61
	**Data set 3**												
		Java (Windows)	23.96	0.0046	0.0098	0.0018	0.0049	0.0008	0.2923	11.13	283.79	36.25	2.24
		Flash (Windows)	22.11	0.0142	0.0186	0.0005	0.0144	0.0002	21.1775	10.42	18.72	10.13	0.06
		HTML5 (Windows)	23.19	0.0008	0.0151	0.0005	0.0008	0.0002	1.9772	12.37	1.43	60.10	0.71
**Experiment 2**													
	**Data set 1**												
		Java (Windows)	29.87	0.0073	0.0142	0.0015	0.0077	0.0010	0.3446	10.76	271.21	32.25	1.92
		Flash (Windows)	27.76	0.0144	0.0193	0.0004	0.0146	0.0001	26.7071	10.24	16.20	10.21	0.05
		HTML5 (Windows)	28.58	0.0009	0.0133	0.0006	0.0008	0.0002	2.4036	13.46	1.09	59.30	0.56
	**Data set 1**												
		Java (macOS)	28.93	0.0132	0.0189	0.0028	0.0133	0.0002	0.3483	10.27	—^c^	—	1.42
		Flash (macOS)	27.76	0.0207	0.0232	0.0002	0.0191	0.0006	21.4324	9.98	13.59	10.07	0.07
		HTML5 (macOS)	29.04	0.0011	0.0194	0.0009	0.0012	0.0002	2.6352	12.31	0.9285	60.20	0.54
	**Data set 1**												
		Java (Linux)	19.61	00113	0.0177	0.0052	0.0104	0.0002	0.4398	8.49	77.84	19.33	1.68
		Flash (Linux)	27.75	0.0291	0.0513	0.0003	0.0325	0.0004	16.3715	9.98	15.12	9.26	0.11
		HTML5 (Linux)	33.22	0.0021	0.0414	0.0008	0.0019	0.0004	3.7369	13.95	1.39	58.7	0.37
**Experiment 3**													
	**Data set 1**												
		Java (LAN)	29.87	0.0073	0.0142	0.0015	0.0077	0.0010	0.3446	10.76	271.21	32.25	1.92
		Java (WAN)	311.47	0.0071	0.0153	0.0011	0.0072	0.0010	0.3417	169.89	268.65	32.23	1.89
	**Data set 2**												
		Flash (LAN)	26.97	0.0148	0.0189	0.0007	0.0149	0.0002	27.5514	10.35	17.59	9.91	0.05
		Flash (WAN)	464.05	0.0145	0.0191	0.0006	0.0148	0.0003	27.4085	176.42	69.18	9.17	0.05
	**Data set 3**												
		HTML5 (LAN)	23.19	0.0008	0.0151	0.0005	0.0008	0.0002	1.9772	12.37	1.43	60.10	0.71
		HTML5 (WAN)	377.51	0.0009	0.0162	0.0008	0.0008	0.0006	2.0147	186.39	1.31	60.02	0.72

^a^s: seconds.

^b^fps: frames per second.

^c^Not tested in macOS.

### Experiment 2: Performance on Multiple Platforms

The average performances of all the functions are presented in Table 4 (Experiment 2). It can be observed from this table that although the 2D performances of Java-, Flash-, and HTML5-based apps running on multiple platforms (Windows, macOS, and Linux) were almost the same, there are obvious differences in 3D performance. Owing to the facts that Java3D is obsolete and the configuration in macOS was much more complicated than expected, surface rendering by Java was not tested in macOS but only tested in Windows and Linux. In terms of the 3D performance on different platforms, Java-based apps on Windows achieved better performance than on Linux and macOS. However, Flash- and HTML5-based apps demonstrated consistent performance across different platforms.

### Experiment 3: Performance based on LAN and WAN

The average performances for each function are presented in Table 4 (Experiment 3). The results of the performances of Java-, Flash-, and HTML5-based apps over LAN and WAN revealed that there was little difference between the LAN and WAN, except for downloading. Given that the data transmission speed over the WAN was lower than that over the LAN, the downloading time was different, as expected. After downloading data to the client computer, the app performance over the WAN was the same as that over the LAN.

### Summary

The experimental results demonstrated that Java-, Flash-, and HTML5-based apps have the ability to yield real-time performances for all 2D functions. However, the 3D performances differed between the 3 apps. In terms of software-based volume rendering, the Java-based app had the highest frame rate; however, it required the longest amount of time for surface rendering and failed to run surface rendering in macOS. In terms of surface rendering, the HTML5-based app had the fastest rendering and the highest speed for fly-through without platform dependence. However, the frame rate of software-based volume rendering by HTML5 was slightly lower than that by Java. The 3D performances of the Flash-based app were worse than both of the other apps.

### Pilot Trial and Feedback From Radiologists

The results of radiologists’ responses are presented in Table 5.

Most radiologists were satisfied with the functions that we provided. However, they were not satisfied with 3D functions in Java and Flash. Three radiologists reported that Java took a long time for surface rendering and Flash provided a significantly low frame rate for volume rendering. For question 8, every radiologist chose HTML5, which means that HTML5 obtained the highest satisfaction among these 3 technologies.

**Table 5:** Radiologists’ responses to the questionnaires.

Radiologist	Question score^a^							
	1	2	3	4	5	6	7	8^b^
Radiologist 1	5	5	5	5	5	5	5	3
Radiologist 2	4	5	5	5	3	3	4	3
Radiologist 3	5	5	5	5	4	4	5	3
Radiologist 4	5	5	5	5	4	4	5	3
Radiologist 5	5	5	5	5	5	5	5	3
Total score	24	25	25	25	21	21	24	N/A^c^

^a^From 1 (strongly disagree) to 5 (strongly agree).

^b^Options: 1 (Java), 2 (Flash), 3 (HTML5).

^c^N/A: not applicable.

## Discussion

### Principal Findings

Currently, there are 3 main technologies for the development of web-based medical imaging apps, namely, Oracle Java, Adobe Flash, and HTML5. Around the 2000s, Oracle Java was a popular internet technology in the field of medical imaging [7-12,14,24]. Since 2010, Flash-based imaging apps have appeared, owing to the ubiquity and small size of the Flash Player [13,16]. Since the release of the World Wide Web Consortium HTML5 recommendation in 2014, there has been a growing trend toward the utilization of HTML5 in the development of medical imaging apps. McLaughlin et al [25] developed a digital training platform for interpreting radiographic images based on HTML5. Their platform had 2 tools, a search strategy training tool and an eye tracking tool, which were used to clarify the image interpretation process [25]. Gorgbjerg [26] presented an HTML5-based web app that could be manipulated as in a picture archiving and communication systems. Zhang [27] created a network-based medical data rendering and sharing system with a client app that was developed by HTML5. This client app has the ability to deliver real-time visualization on the web. Additionally, our previous study [15] provided an evaluation of HTML5 for medical imaging apps and demonstrated that HTML5 can provide an excellent remote access medical imaging experience.

In this study, 3 technologies were used to develop a comprehensive medical imaging app and to evaluate the performances of these technologies in the field of radiology. In terms of accessibility, both Java- and Flash-based apps require a browser plug-in. Despite the fact that the Flash Player has long been one of the most popular browser plug-ins, Apple decided to stop bundling Flash Player in macOS in 2010. Thus, for this group of users, to be able to run Flash-based apps, they must initially install Flash Player. Similarly, to be able to run Java, Java Virtual Machine must be installed. HTML5 does not suffer from this problem because it is the native language used in all browsers. Therefore, HTML5 requires no preinstallation and is a platform-independent technology that provides a high level of accessibility. However, the advantages associated with HTML5 exist only in the latest version of browsers. Older browser versions, such as Microsoft Internet Explorer 8 (or older versions), Mozilla Firefox 3.5 (or older versions), and Google Chrome 10 (or older versions), are not compatible with HTML5. In these cases, users would be required to update their browsers, otherwise, HTML5-based apps could not be launched in their browsers. Furthermore, browsers vary in their level of support for the HTML5 standard, and thus, this leads to inconsistent user experiences. For example, the implementation of the mouse wheel event is different among Internet Explorer, Chrome, Safari, and Firefox. In the case of Chrome, when the mouse wheel is rolled up, the value increases however, in Firefox, the value decreases.

In terms of functionality, all 3 technologies can realize the necessary functions for remote viewing of radiological images. Image processing, such as zooming and windowing, can be provided by all 3 technologies on all platforms. However, implementation of 3D visualization is more complicated than the implementation of image processing, especially for Oracle Java. Oracle Java realizes 3D surface rendering by depending on Java3D API. However, this API has not been updated since 2008. Hence, some problems emerge in recent implementations of Java3D (eg, the Java3D app failed to launch in macOS). 3D visualization by Adobe Flash and HTML5 can be successfully implemented, regardless of the platform. However, it should be noted that Adobe will terminate its support for Flash at the end of 2020. Thereafter, Flash 3D API and Flash Player will not be updated. In this case, only HTML5 has an advanced API and hence can provide a higher level of functionality (compared to Oracle Java and Adobe Flash).

In terms of usability, the experimental results reveal that all 3 technologies can provide 2D image processing on all platforms. However, the 3D performances of these technologies are different. Among these technologies, HTML5 presents the best surface rendering performances in terms of rendering time and frame rate. In terms of volume rendering, HTML5 is not good at software-based volume rendering. However, when integrated with a GPU, HTML5 can provide fast hardware-based volume rendering [28,29].

In terms of interoperability, Oracle Java, Adobe Flash, and HTML5 are designed for developing rich web apps. Therefore, all 3 apps can be connected to a large teleradiology system and placed as client components. Moreover, the source code of HTML5 is exposed online, and therefore, the locations of image data sets can be easily assessed. The source codes of Java and Flash are hidden (inside .JAR and .SWF files, respectively), which prevents unauthorized access to image data sets. Thus, Java and Flash outperform HTML5 in data privacy.

Recently, cloud computing has been used in the field of medical imaging for high-capacity storage, sharing, and intensive computational tasks [30,31]. In this infrastructure, the image data and complex processing tasks are moved from user computers to the cloud. The users then launch an app to access the cloud. In this case, a radiologist can implement the cloud-based medical image analysis using a personal computer from any location. Furthermore, web technology supports the development of the client app in the cloud-based system. With its development, the client app can become more powerful than before. Among these web technologies, HTML5 can develop a zero-footprint web viewer, which requires zero plug-ins, zero latency, and zero maintenance. Therefore, most commercially web-based DICOM (Digital Imaging and Communications in Medicine standard) viewers, such as Ambra [32], medDream [33], and boxDicom [34], switched to an HTML5-based solution recently. All of them can be integrated into any picture archiving and communication systems system. Additionally, medDream provides 3D features, such as maximum intensity projection and 3D rendering, in a browser. We confirmed that their HTML5 solutions can implement necessary interpretation tools, such as 2D image processing and 3D visualization, inside the client browser with satisfactory performance.

Although web technology enables remote viewing of radiological images easily and efficiently, there are still 4 issues affecting current web-based medical imaging apps. First, when data are transmitted over the internet, security is the biggest challenge, and this has encouraged many researches to find ways to keep medical images safe and confidential [35,36]. Second, remote viewing of radiological images is heavily dependent on the network. When internet connections are slow or unavailable, our web apps cannot work properly; hence, network condition is an important factor in teleradiology settings. Third, the specifications of a personal computer are usually inferior to those of dedicated workstations, and therefore, intensive computational tasks, such as volume rendering, cannot be implemented on a personal computer. In our implementation of volume rendering, the rendering region was reduced in order to provide fast volume rendering. Therefore, some interpretation tools need to be customized and simplified in web-based apps. Furthermore, typical medical image sizes range from 512×512×8 bits up to 1024×1024×12 bits. For some imaging apps, the resolution is even higher. Therefore, the client’s screen should support higher resolutions, otherwise the medical images cannot be properly displayed.

### Limitations

Our study and the web-based apps that were developed also have some limitations. First, we were able to obtain feedback from 5 radiologists to conduct a pilot testing, but we were not able to conduct a large and comprehensive investigation on users’ opinions. Therefore, the feedback of users may contain deviations due to a small sample size. Second, only 2 personal computers, one on which Windows and Linux were installed, and another on which macOS was installed, were used in our experiments. The performances of apps may be affected by the hardware specifications. In future, upgraded computer hardware could enhance the performance of our apps.

### Conclusion

Based on the review of existing literature, it is apparent that there is a lack of studies on the evaluation of different internet technologies for remote viewing of radiological images. In this study, 3 main internet technologies (ie, Oracle Java, Adobe Flash, and HTML5) were used to develop comprehensive web-based medical imaging apps. Experiments were conducted to compare these technologies in terms of accessibility, functionality, and usability. Moreover, advantages and disadvantages were discussed. Our research demonstrated that HTML5 is a promising technology for remote viewing of radiological images and can provide excellent performance without requiring any plug-ins. Therefore, our research provides an important reference for future development of web apps in the field of medical imaging, and it could help to identify an optimal solution for remote viewing of radiological images.

## Appendix

Multimedia Appendix 1 Questionnaire.

## Abbreviations

CT: computed tomography
DICOM: Digital Imaging and Communications in Medicine standard
GPU: graphics processing unit
HTML: hypertext markup language
LAN: local area network
WAN: wide area network
