# Supplementary material for: Web-Based Technology for Remote Viewing of Radiological Images: App Validation
Source: J Med Internet Res. 2020 Sep 25;22(9):e16224. doi: 10.2196/16224 (PMC7547396; doi:10.2196/16224)
Supplement: Multimedia Appendix 1 [file jmir_v22i9e16224_app1.docx]

**MULTIMEDIA APPENDIX 1**

Questionnaire

1. The functions of the three applications meet your needs.

A strongly disagree, B disagree, C unsure, D agree, E strongly agree

1. You are satisfied with the 2D functions of the Java-based application.

A strongly disagree, B disagree, C unsure, D agree, E strongly agree

1. You are satisfied with the 2D functions of the Flash-based application.

A strongly disagree, B disagree, C unsure, D agree, E strongly agree

1. You are satisfied with the 2D functions of the HTML5-based application.

A strongly disagree, B disagree, C unsure, D agree, E strongly agree

1. You are satisfied with the 3D functions of the Java-based application.

A strongly disagree, B disagree, C unsure, D agree, E strongly agree

1. You are satisfied with the 3D functions of the Flash-based application.

A strongly disagree, B disagree, C unsure, D agree, E strongly agree

1. You are satisfied with the 3D functions of the HTML5-based application.

A strongly disagree, B disagree, C unsure, D agree, E strongly agree

1. Which one would you rank the highest in terms of overall satisfaction

A Java, B Flash, C HTML5
